# Pediatric Treatment of Anterior-Upper-Single Dental Crossbite Using a Versatile Sagittal Screw System: A Case Series

**DOI:** 10.3390/pediatric17010011

**Published:** 2025-01-21

**Authors:** Antonino Lo Giudice, Alessandro Polizzi

**Affiliations:** Department of General Surgery and Medical-Surgical Specialties, University of Catania, Policlinico Universitario “Gaspare Rodolico-San Marco”, Via Santa Sofia 78, 95123 Catania, Italy

**Keywords:** crossbite, malocclusion, anterior crossbite, interceptive orthodontics, pediatric dentistry

## Abstract

Background/Objectives: Anterior crossbite is characterized by a reverse sagittal relationship between the maxillary and mandibular incisors. Early treatment of an anterior crossbite is advocated to avoid periodontal and traumatic consequences on lower dentition along with growth disturbances in the anterior maxilla and mandible. The present case series describes the usage of a sagittal expansion screw integrated with a removable maxillary plate or fixed appliance to correct an anterior upper single dental crossbite and outlines the clinical rationale and the protocol associated with this appliance system. Methods: A total of four children presenting anterior crossbite were treated using a sagittal expansion screw integrated into a removable plate or a fixed appliance. Results: All patients were successfully treated in less than 4 months. Overjet and overbite were normalized by the end of treatment. The orthodontic treatment received positive feedback from parents and caregivers, who seek a rapid improvement in their children’s aesthetics and function. No significant discomfort or speech difficulties were reported by the parents or patients. Conclusions: The present case series would suggest that a sagittal expansion screw, integrated into both a removable and fixed appliance, can represent a valuable and versatile treatment option for correcting an anterior crossbite.

## 1. Introduction

Anterior crossbite is a malocclusion characterized by one or more mandibular incisor(s) positioned more anteriorly than a maxillary incisor(s) [1,2]. This condition is classified as a skeletal crossbite when it is related to a skeletal discrepancy (maxillary retrognathia and or mandibular prognathism) [3]. A skeletal crossbite is characterized by a concave profile of both the skeletal structure and soft tissues, often necessitating more comprehensive treatments for effective management. It is classified as a dental crossbite if it is the result of a palatal displacement of a maxillary tooth, often associated with the labioversion of the mandibular tooth [4] (Table 1). An accurate differential diagnosis is required to discriminate both conditions and establish the appropriate treatment plane [2].

Dental anterior crossbites have a prevalence between 1.6% and 8% and typically becomes apparent during the early mixed dentition phase [5,6]. The reported prevalence could also depend on the age of the child, ethnic group, and the methods of registration [7]. Several etiological factors can contribute to the abnormal eruption pattern of upper incisors in these patients: trauma to primary incisors leading to permanent tooth bud displacement, delayed exfoliation of primary teeth resulting in palatal positioning of erupting incisors, a cleft lip, supernumerary teeth, odontomas, incisor crowding, or habits such as lower lip biting [8,9,10]. If left untreated, dental crossbite may include gingival recession, loss of alveolar bone support, and mobility of lower incisors, along with potential growth disturbances in the anterior maxilla [11]. As a consequence, early treatment of an anterior crossbite is advocated in order to correct the position of the affected maxillary teeth to establish a stable overbite relationship.

An anterior crossbite is often detected early at pediatric age due to its aesthetic implications, prompting parents to seek orthodontic consultations for their children. Since compliance is particularly critical at this age, the orthodontic system used to correct anterior crossbite should be comfortable and well-tolerated to ensure effectiveness and efficiency at the same time. Different methods have been described in the literature, firstly orthodontic fixed appliances. Although fixed orthodontic appliances require less patient compliance they are linked to time-related side effects, such as the development of white spots, root resorption, and potential negative effects on the oral health-related quality of life of patients [12,13]. In this regard, removable appliances, or bite planes [11,14] have been proposed in the literature, each with its own advantages and disadvantages in terms of clinical management [15,16,17].

The present paper describes the usage of a sagittal expansion screw integrated with a removable maxillary plate or fixed appliance to correct an anterior dental crossbite and outlines the clinical rationale and the protocol associated with this appliance system.

## 2. Case Series

### 2.1. Case 1

**Figure 1:**
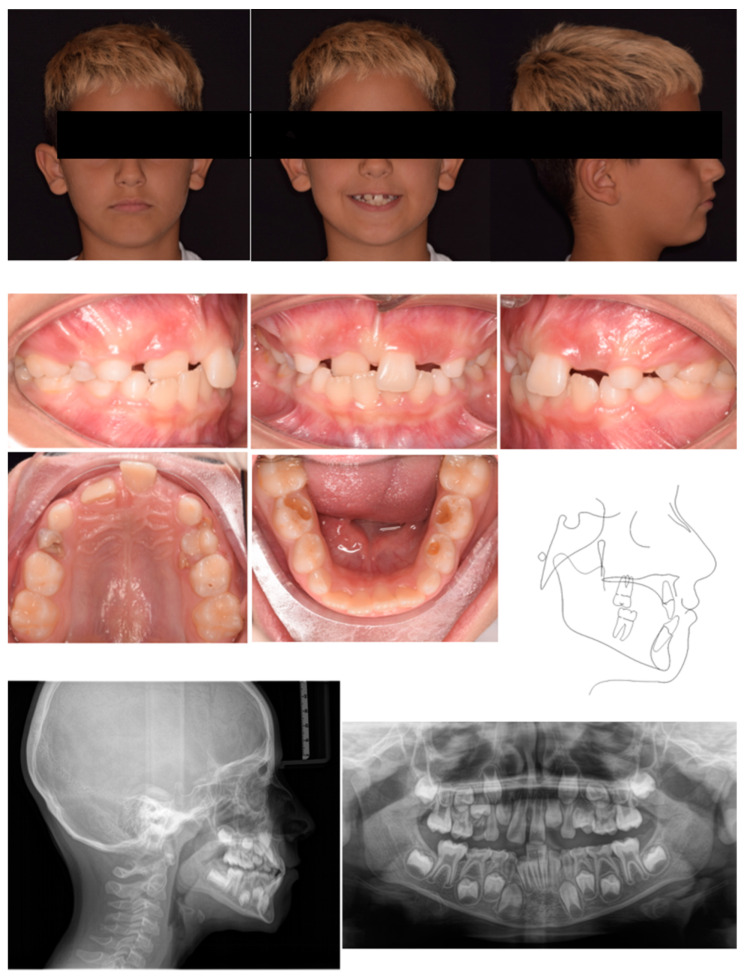
Case 1 at baseline.

A 7-year-old male was referred by the pediatric dentists for orthodontic consultation at a private practice. The medical history was reviewed and no specific pathology was reported. The parents reported as the chief complaint the poor visibility of the right central incisor and the excessive exposure of the upper left central incisors, which caused the appearance of having only one central incisor while smiling. This condition exposed the child to bullying by his peers. Extra-oral examination showed an anterior crossbite of 11, a vestibular inclination of 21, and the absence of upper lateral incisors (Figure 1). Intraoral analysis showed neutral occlusion, crossbite 11–41, and proclination of tooth 21 likely resulting from strength anterior contact with lower incisors that occluded between 11 and 21. Also, the ectopic position of 11 represented an obstacle to the eruption of 12. The patient presented radicular residuals of tooth 64 along with extended carious lesions on teeth 5.4, 7.5, 76, and 85 (Figure 1). The radiographic evaluation confirmed the impaction of tooth 1.2 (Figure 1). Cephalometric analysis revealed a Class I skeletal relationship with a negative overjet and an increased inter-incisal angle, caused by the abnormal positioning of the upper incisors, indicating a dental anterior crossbite (Figure 1). The treatment plan comprised two phases: an initial phase involving pedodontic treatments, followed by interceptive orthodontic treatment to correct the anterior crossbite.

**Figure 2:**
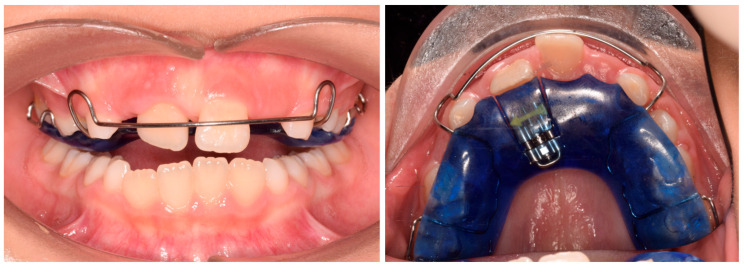
Customized removable maxillary appliance.

Phase 1 included the extraction of tooth 54 and the residual root of tooth 64, the removal of carious lesions on teeth 65, 74, and 85 followed by composite restorations, and indirect pulp capping on tooth 75 followed by composite reconstruction (after 30 days). Phase 2 started after pedodontic treatments. In particular, intraoral scans were registered and a removable maxillary appliance was designed featuring the following characteristics: posterior bite planes to facilitate anterior disclusion, expansion screw with antero-posterior orientation, selective resin cut on tooth 11, vestibular arch with dedicated loops (Figure 2). The activation protocol consisted of biweekly adjustments (2 × 0.10 mm), with full-day wear required except during meals and oral hygiene procedures. Also, the vestibular arch was contracted by slightly closing the lateral loops to favor the crown lingual inclination of 21.

**Figure 3:**
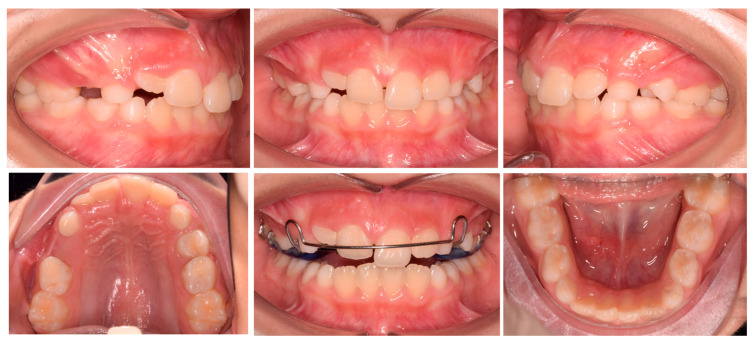
Case 1 improvement.

**Figure 4:**
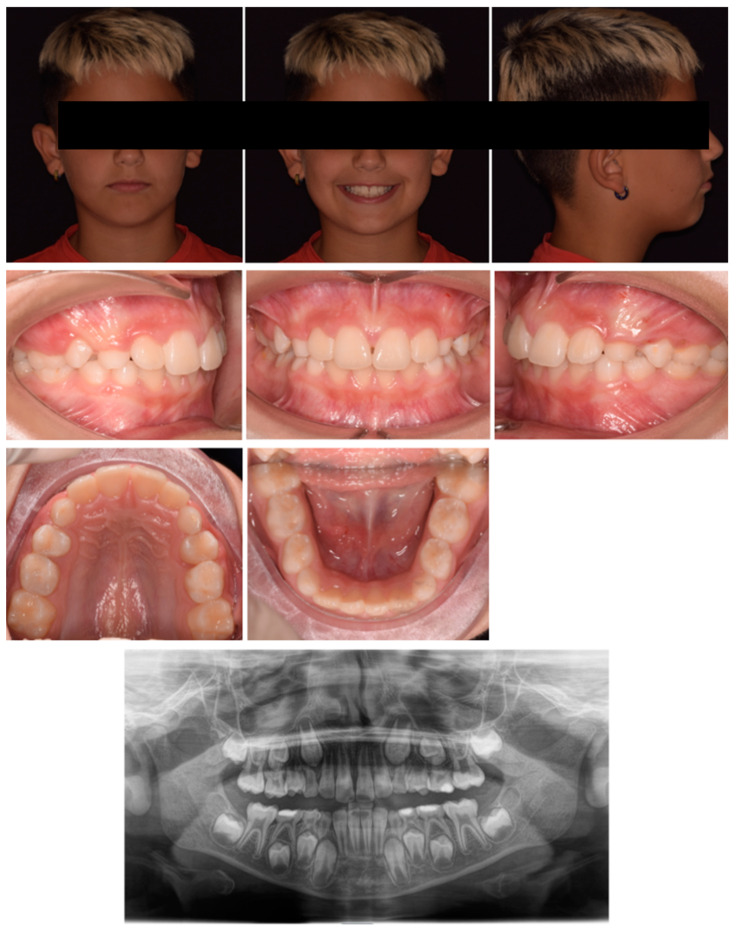
Twelve months after treatment.

After three months, there was an evident correction of the crossbite and spontaneous eruption of 12. Once the correct inclination of 11 was achieved, the device was utilized as a nocturnal space maintainer (Figure 3). A total of twelve months after treatment the results were stable with a significant improvement in the smile aesthetic (Figure 4). Tooth 12 spontaneously completed the eruption process. No significant discomfort or speech difficulties were reported by the parents or patients. The panorex showed no signs of root resorption or periodontal damage on 11. The cephalometric radiograph was not performed in accordance with the A.L.A.D.A. and A.L.A.D.A.I.P. principles [18,19].

### 2.2. Case 2

**Figure 5:**
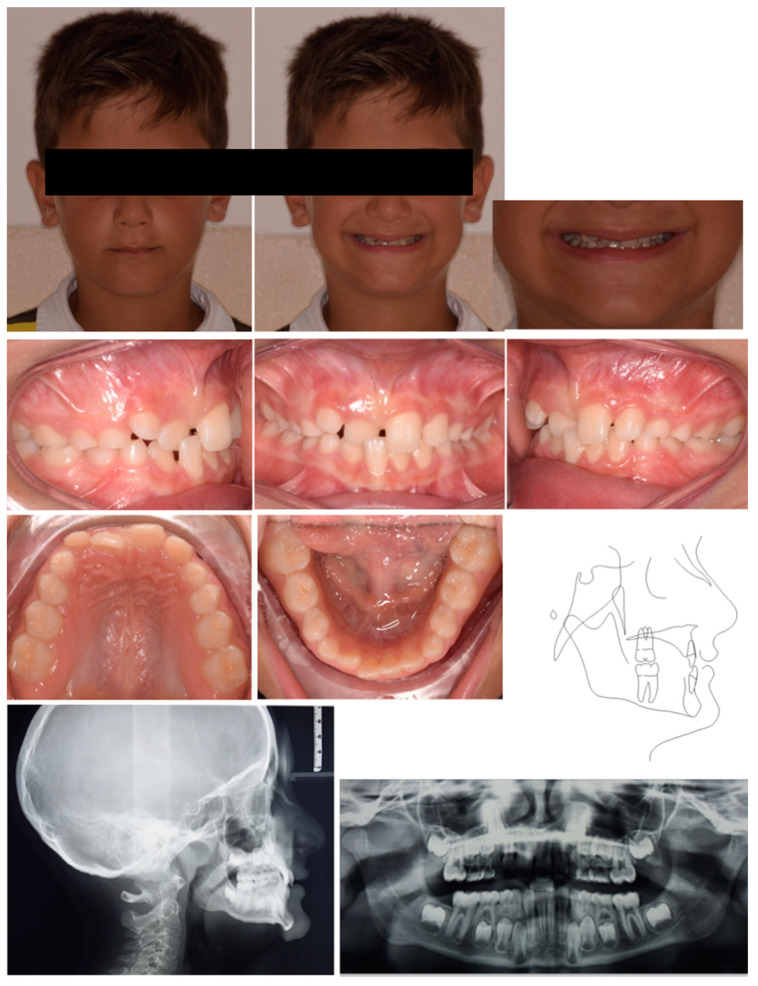
Case 2 at baseline.

An 8-year-old male was referred by the pediatric dentists at a private practice for orthodontic evaluation due to parental concern regarding the retro-position of the right central incisor, while the child reported tension and pain in the incisor region while biting. The medical history was reviewed and no specific pathology was reported. Extraoral examination revealed the anterior crossbite of 11, although the poor teeth exposure at smiling mitigated the unaesthetic effect caused by the ectopic incisor position (Figure 5). The intraoral assessment showed a neutral occlusion, anterior crossbite 11–41, normal position of 21, and buccal inclination 41 with gingival recession and loss of attached gingiva (Figure 5). The panorex showed no evidence of carious or inflammatory lesions (Figure 5). Cephalometric analysis indicated a Class I skeletal relationship with a slight tendency towards Class III (primarily due to a morphological prominence of the symphysis), negative overjet, increased inter-incisal angle, and poor bone support of 41. All these parameters were consistent with the diagnosis of anterior dental crossbite (Figure 5).

**Figure 6:**
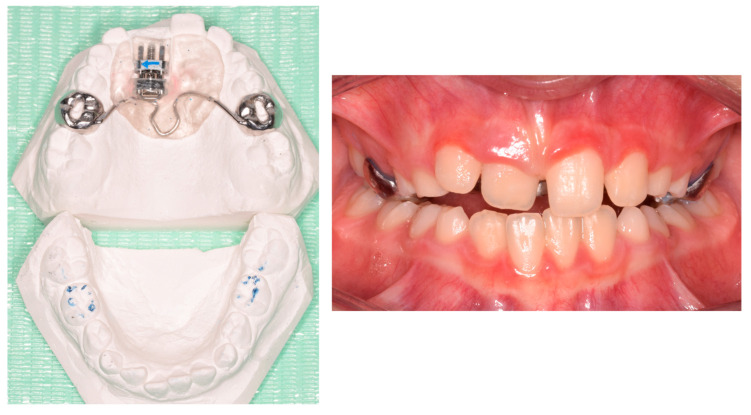
Customized palatal bar with an anterior resin pad equipped with an expansion screw.

The proposed treatment plan included a palatal bar featuring posterior bite planes to facilitate anterior disclusion, along with an anterior resin pad equipped with an expansion screw oriented in the anteroposterior direction, with selective resin cut on tooth 11 (Figure 6). Posterior bite ramps were designed onto the molar bands using CAD software, which was subsequently produced using laser melting technology. The design of the ramps followed the anatomical contours of the lower molar cusps, ensuring optimal stabilization of the bite without the risk of unstable contacts. The height of the ramps was determined by registering the desired posterior disclusion. The activation protocol consisted of biweekly adjustments (2 × 0.10 mm). Once the correct position of 21 was achieved, the device was removed.

**Figure 7:**
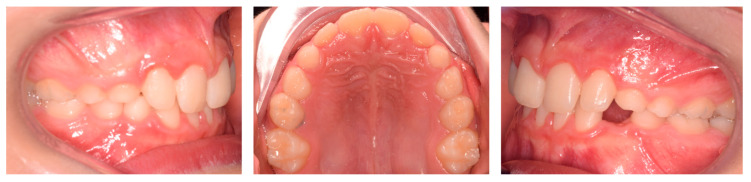
Case 2 after four months.

Four months after treatment, complete resolution of the crossbite was observed, along with significant improvement in the periodontal condition of 41 (Figure 7). The cephalometric radiograph was not conducted in accordance with A.L.A.D.A. and A.L.A.D.A.I.P. principles [18,19].

### 2.3. Case 3

**Figure 8:**
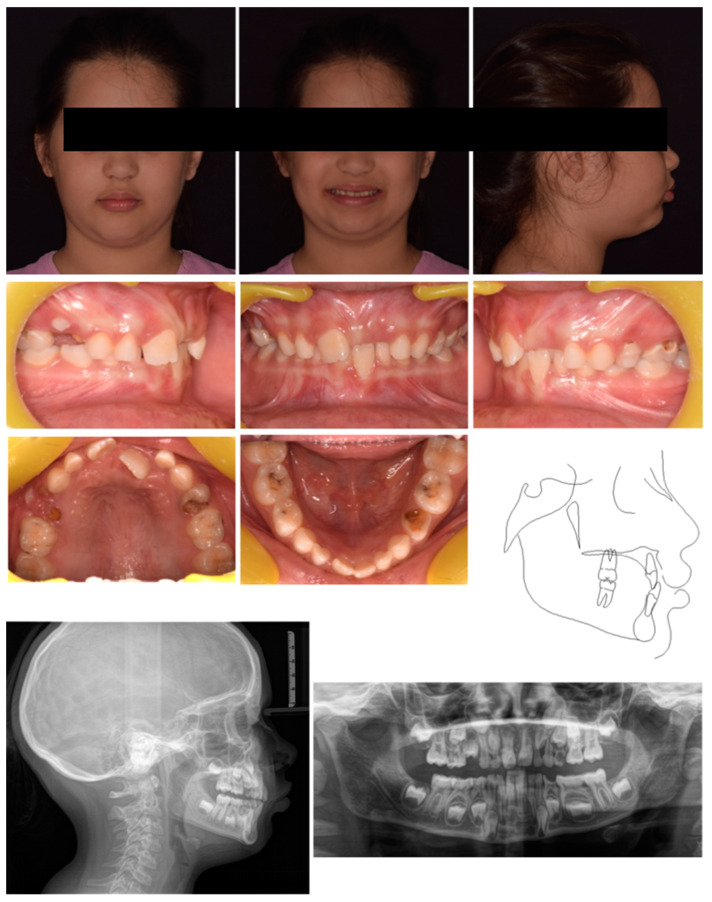
Case 3 at baseline.

A 7-year-old female was referred by the pediatric dentists at a private practice for orthodontic consultation due to an unaesthetic smile. The medical history was reviewed and no specific pathology was reported. The parents reported as the chief complaint the poor visibility of the upper left central incisor and the excessive exposure of the upper right central incisors; the patient also reported pain at the incisor region during biting. Extra-oral examination showed poor visibility of 21, although the poor teeth exposure at smiling mitigated the unaesthetic effect caused by the ectopic incisor position (Figure 8). Intraoral analysis showed neutral occlusion, crossbite between 21 and both 31–72, severe proclination of 31 with gingival recession and loss of attached gingiva, and a severe deep bite. The patient also featured poor oral hygiene, extensive carious lesions on teeth 55, 64, 65, 74, 75, 84, 85, and radicular residual of tooth 54 (Figure 8). The radiographic evaluation confirmed the extent of the carious lesions and showed radiographic crowding on the upper-left posterior region (Figure 8). Cephalometric analysis revealed a Class I skeletal relationship with a slight tendency toward Class II pattern, negative overjet, and an increased inter-incisal angle attributable to the abnormal positioning of the upper incisors. All these parameters confirmed the diagnosis of a dental anterior crossbite (Figure 8). The treatment plan comprised two phases: an initial phase involving pedodontic treatments, followed by interceptive orthodontic treatment to correct the crossbite.

Phase 1 included the extraction of teeth 54 and 64, treatment of carious lesions on teeth 75 and 84 followed by composite restorations, indirect pulp capping on teeth 55, 65, and 85 followed by composite reconstruction (after 30 days), pulpotomy, and restoration with vetro-ionomeric cement on tooth 74.

**Figure 9:**
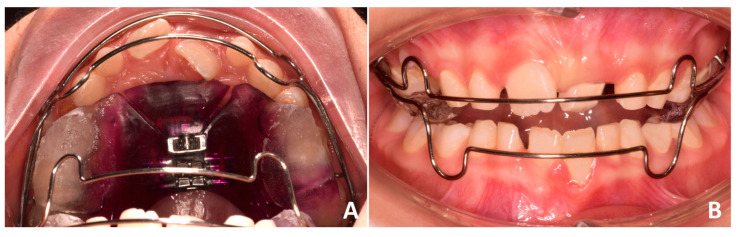
Customized removable maxillary appliance. (**A**) occlusal view; (**B**) frontal view.

Phase 2 started after pedodontic treatments. In particular, the intraoral scans of the patient were acquired, and a removable maxillary appliance was designed with the following characteristics: posterior bite planes to facilitate anterior disclusion, a sagittal expansion screw, and a selective anterior fan-shape resin cut. The resin cut is designed to apply the force on the disto-lingual surface of the 21 during screw activation, generating a force moment that would favor the mesial rotation of the tooth; the control of the planned rotational movement was also supported by the contact between the vestibular arch and the mesio-vestibular surface of the same tooth. A lower vestibular arch was also included in the appliance to provide retro-inclination of the 31 by closing the loops bilaterally. The activation protocol consisted of one activation per week (1 × 0.25 mm), with full-day wear required except during meals and oral hygiene procedures (Figure 9A,B).

**Figure 10:**
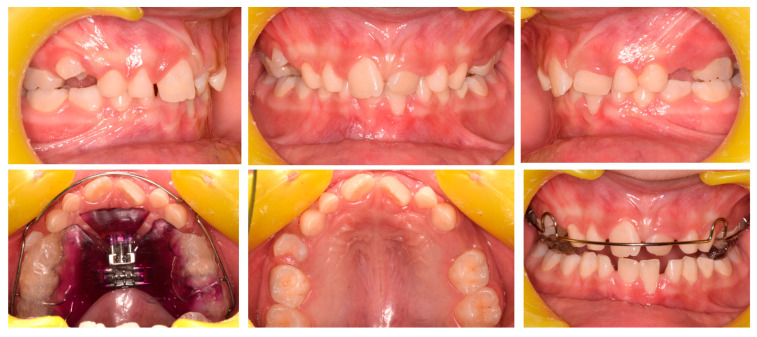
Case 3 after four months.

**Figure 11:**
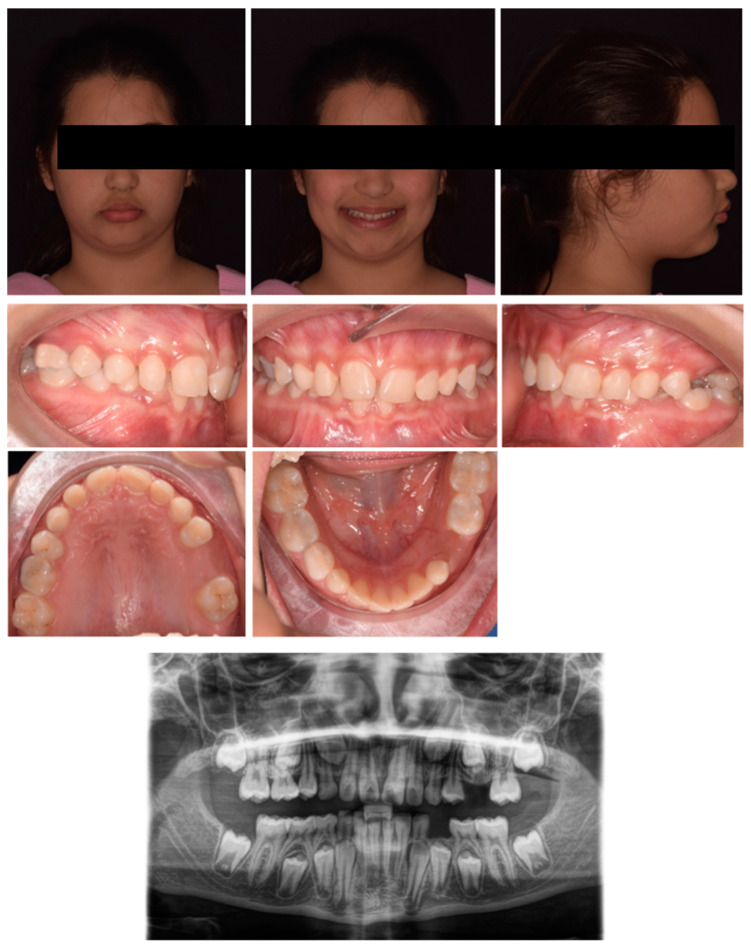
Case 3 after ten months.

After four months, there was an apparent correction of the anterior crossbite. However, the device was activated even after the crossbite correction to facilitate the derotation of the 21 (Figure 10). A total of ten months after treatment, complete resolution of the crossbite was observed, with spontaneous eruption of tooth 2.1, improvement of the overbite, and significant aesthetic improvement of the smile (Figure 11). The rotation of the 2.1 was not fully achieved; however, the primary therapeutic objective was to eliminate occlusal interferences, and the completion of alignment could be deferred to the treatment with fixed appliances. No speech difficulties were reported by the parents or patient, but the patient reported the transient discomfort of perceiving only posterior contact “with the mouth still opened” due to the presence of posterior bite planes. The panorex confirmed the absence of additional carious lesions or periapical inflammation; teeth 6.5 and 7.4 were extracted due to the recrudescence of carious lesions. A cephalometric radiograph was not performed in accordance with the A.L.A.D.A. and A.L.A.D.A.I.P. principles [18,19].

### 2.4. Case 4

**Figure 12:**
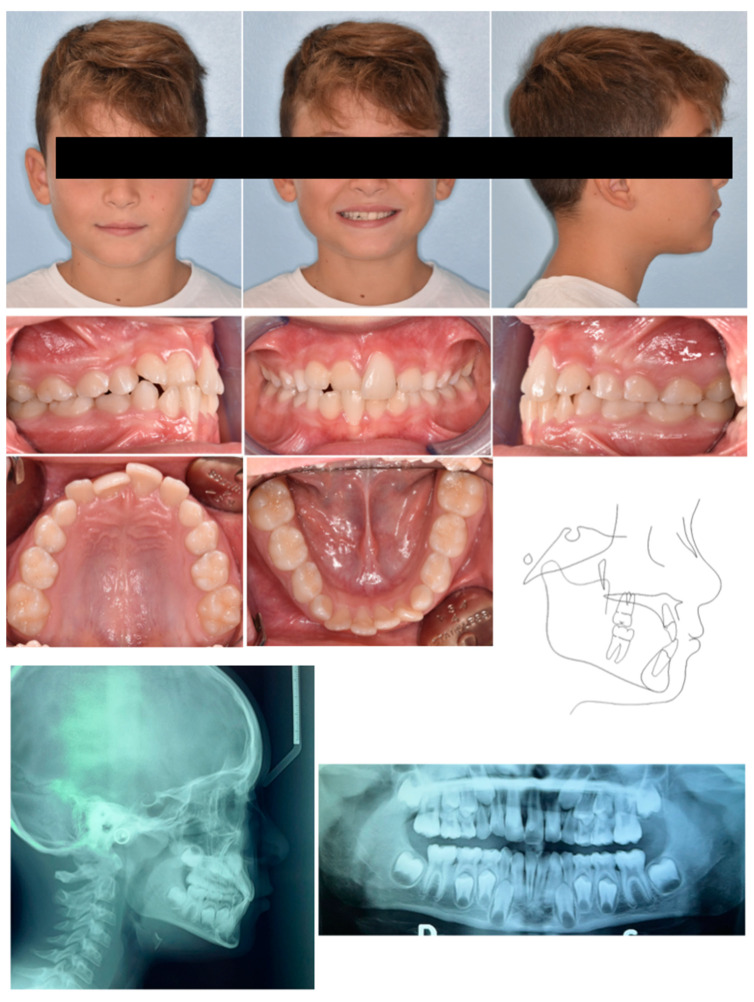
Case 4 at baseline.

A 7-year-old male was referred by the pediatric dentist for orthodontic consultation at a private practice due to an unaesthetic smile. The medical history was reviewed and no specific pathology was reported. In particular, the parents noted the poor visibility of the right central incisor and the excessive exposure of the upper left central incisors while smiling; the patient also referred to strong occlusal contact and pain at the incisor region during biting. Extra-oral examination showed scarce exposure of 11 and a good concordance between the lower lip and smile curve (Figure 12). Intraoral examination showed molar Class I relationship, crossbite 11–41, altered morphology of 21 with gingival defect, upper and lower anterior crowding, proclination of 41 with gingival recession, and loss of attached gingiva. Panorex showed the presence of an interproximal carious lesion on teeth 54, 55, 64, and 65, and radiographic crowding on the upper left posterior region. Cephalometric analysis revealed a Class I skeletal negative overjet, and an increased inter-incisal angle, indicating a dental anterior crossbite. The treatment plan comprised two phases: an initial phase involving pedodontic treatments, followed by interceptive orthodontic treatment to correct the crossbite.

**Figure 13:**
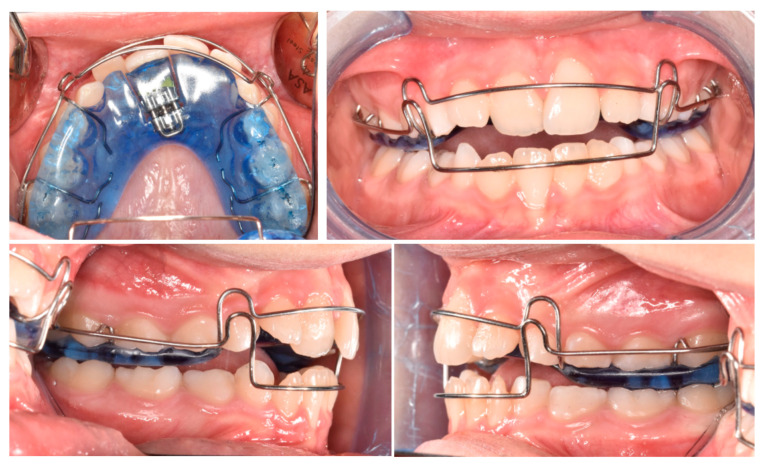
Customized removable maxillary appliance.

Phase 1 included the treatment of carious lesions on teeth 54, 55, 64, and 65 and subsequent composite restoration. After pedodontic treatments, Phase 2 started with intraoral scans and a removable maxillary appliance was designed with the following characteristics (Phase 2, Figure 13): posterior bite planes to facilitate anterior disclusion, an expansion screw with sagittal orientation, and with a selective resin cut on 11. A lower vestibular arch was also included in the upper appliance to simultaneously provide retro-inclination of 41 by slightly closing the loops bilaterally. The activation protocol consisted of biweekly adjustments (2 × 0.10 mm), with full-day wear required except during meals and oral hygiene procedures.

**Figure 14:**
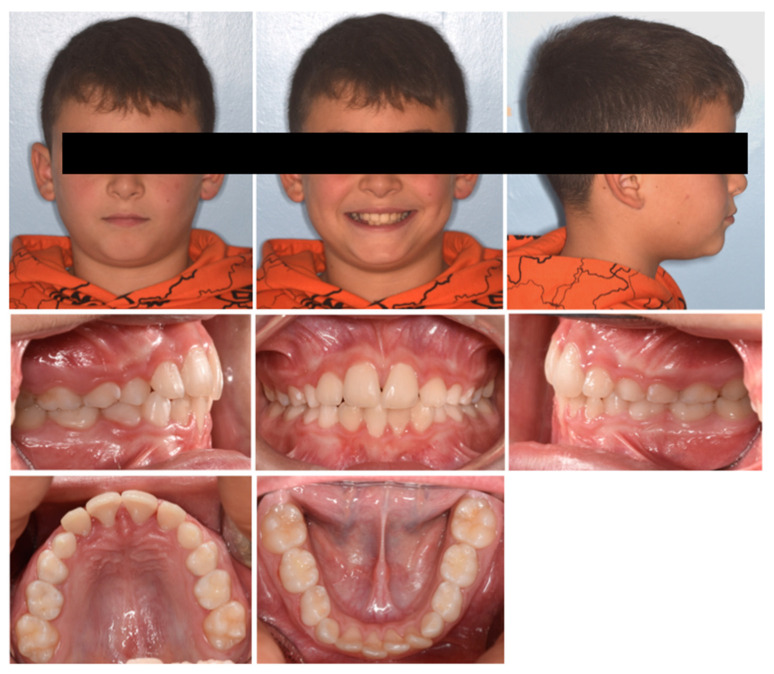
Case 4 after four months.

After four months, there was an evident correction of the crossbite and spontaneous eruption of tooth 11 (Figure 14). Once the vestibulolingual positioning of tooth 11 was achieved, consistent with 21, the device was utilized as retention for 2 months. Occlusal conditions were stable, and significant improvement was observed in the periodontal soft tissue of 41. No significant discomfort or speech difficulties were reported by the parents or patients. No radiographic examinations were required due to the short-term treatment progress.

## 3. Discussion

The primary objective of orthodontic treatment in mixed dentition is to prevent the development of more complex malocclusions and to enhance arch integrity in anticipation of permanent tooth eruption [6]. There is a broad consensus that early intervention for anterior crossbites—regardless of whether they are skeletal, functional, or dental—is crucial to mitigate potential adverse consequences on dentition and craniofacial development [9,20,21].

Concerning a dental anterior crossbite, the effects on the lower dentition are particularly critical, as periodontal damage has been documented in the lower affected tooth, as also shown in Cases 2, 3, and 4 of the present case series. These conditions, if left untreated, may require periodontal treatment in the future. Furthermore, spontaneous or induced (biting) pain, traumatic enamel abrasion, fractures, and mobility of anterior teeth are frequently observed in these patients [9]. Another consequence of an anterior crossbite is that the mandible can be positioned in a functional Class III relationship, which, if left untreated, can significantly affect the kinematics of the temporomandibular joint and the growth of the lower third of the face [22].

The treatment of an anterior crossbite does not demand complex orthodontic biomechanics; rather, it requires a simple vestibular crown tipping movement, achievable with different orthodontic appliances. Generally, the appliances used can be categorized as fixed and removable devices.

A significant advantage of fixed devices is that they do not require patient cooperation.

However, they are associated with time-related adverse effects, such as white spots and root resorption, and are associated with a lower rated impact on patients’ oral health-related quality of life [12]. In this regard, simpler methods that do not require the use of any orthodontic appliances such as bilateral occlusal build-ups (bonded to the mandibular second primary or first permanent molars) or a bonded inclined bite plane (bonded to the incisal margins of lower incisors) have been proposed for correcting an anterior crossbite during mixed dentition [2,11,14]. This approach may be particularly promising, especially for special-needs children.

Although the effectiveness of removable devices is strictly related to patient compliance, they are generally well-tolerated for two reasons: firstly, they allow for better oral hygiene maneuvers; secondly, they do not require bite planes/ramps on single teeth which generate the unpleasant sensation of perceiving few occlusal contacts “with the mouth still opened” when using fixed appliances. Indeed, occlusal bite planes are crucial to avoid occlusal interferences in the correction of the crossbite. Using removable appliances, the bite planes are distributed across the entire posterior-lateral segment (Figure 13), providing better occlusal stability and comfort during the critical phase of reversing the bite, when occlusal contacts are reduced. Table 2 provides clinicians with a summary of the characteristics, advantages, and disadvantages of the appliances reported in the literature, including those discussed in this article.

Concerning the activation system, the use of a sagittal expansion screw can offer a valid alternative to other systems described in the literature. Below is a brief overview of the advantages of this system.

The expansion screw enables selective action and the transmission of calibrated and predefined forces with each activation (0.10 mm or 0.25 mm). Additionally, the programmed movement aligns with the range of movement achieved with clear aligners (0.20–0.30 mm) [27]. As shown in the treated cases, no signs of root resorption or periodontal damage were observed in the post-treatment panorex.

Because the applied force is pre-determined, it allows for predictable additional activation when the objective is to accelerate tooth movement to overwhelm the traumatic interference generated during the critical stage of bite reverse (when the patient is not wearing the appliance). In this regard, a 0.10 mm screw provides greater versatility, avoiding the application of overloaded forces. During this phase, if a removable device is used, it may be beneficial to prescribe a semi-liquid diet to prevent occlusal overload on the incisors or to eat with the device in situ.

By modifying the resin design, rotational moments can be introduced if necessary.

If lower incisors show signs of periodontal damage, a lower vestibular arch can be integrated into the system to generate a lingual crown movement that accelerates the resolution of the traumatic interference generated during bite reverse (when the patient is not wearing the appliance).

The sagittal expansion screw can be integrated into a fixed system, such as a transpalatal bar. In this case, it is advisable to use CAD bands with integrated posterior bite ramps. The design of these ramps should conform to the anatomical contours of the lower molar cusps, ensuring optimal stabilization of the bite without the risk of deflective contacts.

The small sample size and limited variability in patient characteristics may reduce the generalizability of the findings to broader populations or more complex cases. Additionally, the relatively short follow-up period does not allow for the evaluation of long-term stability or the potential relapse of the treatment outcomes.

## 4. Conclusions

The present case series would suggest that a sagittal expansion screw, integrated into both a removable and fixed appliance, can represent a valuable and versatile treatment option for correcting anterior crossbite. The orthodontic treatment received positive feedback from parents and caregivers, who seek a rapid improvement in their children’s aesthetics and function. Randomized prospective studies are encouraged to compare and evaluate treatment efficiency, side effects, and the impact on patients’ quality of life due to the proposed method compared to those previously reported in the literature.

## Figures and Tables

**Table 1:** Key Characteristics and clinical implications of an anterior crossbite.

Aspect	Details
Definition	An anterior crossbite is a malocclusion where mandibular incisors are positioned anteriorly to maxillary incisors.
Classification	- Skeletal crossbite: Caused by skeletal discrepancies (e.g., maxillary retrognathia, mandibular prognathism). - Dental crossbite: Due to palatal displacement of maxillary teeth and/or labioversion of mandibular teeth.
Prevalence	Ranges between 1.6% and 8%, typically observed in the early mixed dentition phase.
Etiological Factors	- Trauma to primary incisors - Delayed exfoliation of primary teeth - Cleft lip - Supernumerary teeth, odontomas - Incisor crowding - Habits (e.g., lower lip biting)
Potential Complications	- Gingival recession - Loss of alveolar bone support - Mobility of lower incisors - Growth disturbances in the anterior maxilla
Treatment Rationale	Early treatment is critical to correct tooth positioning and establish a stable overbite relationship.
Therapeutic Options	- Fixed appliances - Removable appliances - Bite planes

**Table 2:** Characteristics of the appliances used to correct an anterior crossbite reported in the literature.

Appliance	Pros	Cons
Quad-elix with lateral arms extended anteriorly to the incisor in crossbite [23]	Compliance not required	More difficult oral hygiene maneuver
	Simultaneous transverse and sagittal correction	
Z-Spring integrated into fixed appliance (transpalatal bar) [24]	Compliance not required	More difficult oral hygiene maneuver
	Selective forces applied on tooth in crossbite	
Z-Spring integrated onto removable appliance (maxillary plate) [25]	Selective forces applied on tooth in crossbite	Compliance required
	Easier oral hygiene maneuver	
Clear Aligners [9]	Selective forces applied on tooth in crossbite	Compliance required
	Versatility in modulating range of movement	
	Correction of other discrepancy in the same treatment plan	High production cost
	Nickel or Metal allergies	
Composite bite plane (anterior or posterior) [26]	Compliance not required	Discomfort
	Cost (No appliance fabrication)	Risk of enamel damage due to occlusal trauma (anterior plane)
		Risk of posterior intrusion (posterior bites)
2 × 4 Fixed Appliance [11]	Compliance not required	Discomfort (bonded bite plane)
	Cost	Required higher orthodontic skills

## Data Availability

Data are contained within the article.
